# Challenges in validating candidate therapeutic targets in cancer

**DOI:** 10.7554/eLife.32402

**Published:** 2018-02-08

**Authors:** Jeffrey Settleman, Charles L Sawyers, Tony Hunter

**Affiliations:** 1 Calico Life SciencesSouth San FranciscoUnited States; 2 Memorial Sloan Kettering Cancer CenterNew YorkUnited States; 3 Salk Institute for Biological StudiesLa JollaUnited States

**Keywords:** reproducibility, melk, protein kinase, scientific publishing, replication

## Abstract

More than 30 published articles have suggested that a protein kinase called MELK is an attractive therapeutic target in human cancer, but three recent reports describe compelling evidence that it is not. These reports highlight the caveats associated with some of the research tools that are commonly used to validate candidate therapeutic targets in cancer research.

Over the past two decades or so, a significant fraction of cancer-related research has been focused on the discovery and validation of candidate therapeutic targets. These efforts have unquestionably yielded findings that have provided a critical foundation for the development of many successful oncology drugs – most notably, a variety of inhibitors of oncogenic kinases and several relatively new immunotherapeutic agents that have already provided clinical benefit to many patients. At the same time, despite the best efforts of the community, a large number of published reports that claim to have discovered a new target fail to achieve a sufficient level of follow-up validation, either pre-clinically or clinically.

Here, we provide some perspective on this important challenge to the field, highlighting as an example the maternal embryonic leucine zipper kinase (MELK). This protein kinase has been implicated in cancer in dozens of publications spanning more than a decade, but its role in cancer has now been called into question by three recent articles in eLife (Lin et al., 2017; Huang et al., 2017; Giuliano et al., 2018). We focus on three topics in particular: the use of cancer cell lines as models for target validation and therapeutic efficacy; the use of RNA interference for target validation; and the use of small-molecule pharmacologic inhibitors.

## Issues related to the use of cancer cell lines

Among the key challenges faced by cancer researchers aiming to validate candidate therapeutic targets are the limitations of the model systems and reagents available to them. Most investigators rely heavily on human cancer-derived cell lines and associated tumor xenografts in mice to examine the role of candidate genes and the efficacy of candidate therapeutics. Such models have several attractive attributes. For example, there are hundreds of such cell lines derived from a variety of tissue types that are commercially available, and their diversity has the potential to capture the increasingly recognized heterogeneity among tumors associated with the real-world patient population. In addition, most of these cell lines have now been genomically annotated, and information regarding mutations, gene expression, and other 'omic' features is now readily accessible through various public databases. And, importantly, these models have proved to faithfully capture at least some elements of target dependency and pharmacologic efficacy that have successfully translated to the clinic (Sharma et al., 2010).

While cell line models have been an invaluable tool for cancer researchers, they are not without problems. Cell line identity issues, which have led to many erroneous published claims, have attracted increasing attention, but can now be effectively managed through STR-based 'fingerprinting' (Freedman et al., 2015). As a result, this is expected to become a less frequent problem with time. However, the 'same' cell line tested in two different laboratories can yield different results – potentially reflecting seemingly minor, but functionally consequential differences in culture conditions (such as media type, serum source, plating density, 2D vs 3D culture, cell line source, and passage history). There is also the epigenetic drift that can arise during long-term passage in culture and can lead to a change in the phenotypic properties of the culture that significantly impacts specific gene dependencies as well as response to therapeutic treatments – even among isogenically-matched lines.

Furthermore, while most investigators now appreciate the importance of testing therapeutic hypotheses in multiple cancer models (owing to disease heterogeneity), findings that emerge from a 'handful' of tested cell lines sometimes fail to extend to a larger panel of models upon broader analysis, especially in cases where a biomarker-response correlation is relatively weak. Platforms that enable such testing on a very large scale (hundreds of cancer cell lines) have emerged only recently, and while such platforms are not yet readily accessible to most researchers, they have the potential to provide important additional contextual information to support therapeutic claims, thereby providing insights to guide prioritization of the most attractive candidate targets emerging from the literature (Garnett et al., 2012).

## Issues related to the use of RNA interference

We now discuss the some of the reagents that are used to model gene dependency and pharmacologic responsiveness. RNA interference, in the form of siRNAs and shRNAs, has been widely used to test the functional requirement for genes of interest in cancer cell models. While this is unquestionably a powerful methodology, it is also widely recognized that it is fraught with the potential to produce misleading results due to off-target effects. Many investigators try to mitigate this problem by using multiple RNAi reagents directed against the same target, or to use 'rescue constructs' to verify the on-target nature of any observed effects. However, the typical assay endpoints that are relevant to cancer cells – such as the inhibition of cell proliferation or the induction of cell death – are especially vulnerable to off-target effects that might impact a large number of functionally important genes. Consequently, the results of such studies can be misleading, even when controls are included.

More recently, CRISPR-based methods to evaluate gene dependency in cultured cells have become increasingly used. This technology, which appears to be less prone to off-target effects, is gradually replacing RNAi methodology for many investigators and could, therefore, eventually reduce the number of spurious findings related to target validation efforts.

## Issues related to the use of small-molecule inhibitors

A second class of reagent – pharmacologic tool compounds, especially 'small-molecule' inhibitors – is widely used for target validation in cancer cells and xenografted tumors. Such compounds, which are widely reported in the cancer literature, and are readily available from a large number of commercial vendors, can be even more problematic than RNAi reagents.

First, the identity and purity of these compounds is often uncertain and highly variable. Thus, a recent report that included analysis of more than 8,500 purchased small-molecule pharmacologic agents revealed that 29% of these compounds did not pass quality control testing (Corsello et al., 2017). Unfortunately, most investigators do not have the means to easily test the quality of these purchased materials and, therefore, they proceed under the assumption that they have received reagents of adequate quality for experimentation.

Second, it is now widely acknowledged that many small-molecule inhibitors can affect multiple cellular proteins, in addition to the protein of interest. In many cases, the identity of the off-target proteins may not even be known. Unfortunately, it is generally difficult to perform an appropriate 'rescue' study to verify the on-target nature of any observed effects of these compounds, consequently leading to a large number of published reports that erroneously attribute the effects of such inhibitors to the wrong target.

The extent to which such reports have misled the cancer research community is unknown, but is likely to be substantial. Notably, there are now many examples of published studies that compellingly demonstrate the off-target effects of putative selective inhibitors. However, despite such reports, investigators continue to publish findings based on the presumption of a selective activity of those very same inhibitors. For example, in 2014 Heinemann et al. reported compelling evidence that a histone demethylase inhibitor called GSK-J1/J4 acted on a number of histone demethylases (Heinemann et al., 2014) and was not, therefore, a selective inhibitor, as had been previously reported (Kruidenier et al., 2012; see also Kruidenier et al., 2014). However, despite this exchange being published in Nature, more than a dozen subsequent publications have described studies that yielded conclusions based on the use of GSK-J1/J4 to selectively target the KDM6/JMJD3 demethylases. Of course, it is also widely recognized that there are very few truly mono-selective pharmacologic small molecules; and yet, many of these have been useful both as research tools and as drugs. But it is important that investigators take appropriate measures to increase the likelihood that any observed effects of these agents reflect their interaction with the intended target or targets.

The extent to which such reports have misled the cancer research community is unknown, but is likely to be substantial.

## MELK as a case study

Having described a few of the factors that challenge our ability to validate targets, let’s consider the case of MELK. This leucine zipper-containing serine/threonine kinase was first reported 20 years ago as a new member of the AMP kinase family whose expression is increased in pre-implantation mouse embryos, implicating MELK in early development (Heyer et al., 1997). In 2005, MELK was first implicated in cancer in a study that reported elevated levels of MELK RNA expression in many human tumors and in a mouse colorectal cancer model, and demonstrated that MELK-targeted RNA-interference inhibited proliferation in cultured human cancer cell lines and also in a xenograft tumor model (Gray et al., 2005). Since that initial article, there have been another 32 articles that specifically implicate MELK as a therapeutic target in human cancer (see column 1 in Table 1), including an article by Wang et al. that was published in eLife in 2014 (Wang et al., 2014).

**Table 1.** Articles suggesting a link between MELK and human cancers. The 33 articles listed in column 1 of this table suggest that MELK has a role in a variety of human cancers, which makes in a candidate therapeutic target for cancer drugs. Columns 2–5 indicate which articles reported certain kinds of evidence to make the link between MELK and cancer, column 6 indicates the studies that used a MELK inhibitor called OTS167 (which is currently undergoing clinical trials). However, three recent papers (Lin et al., 2017; Huang et al., 2017; Giuliano et al., 2018) suggest that MELK should not be considered as a candidate therapeutic target.

	Articles reporting that MELK expression is up-regulated in various human cancers	Articles reporting an association between increased MELK expression and poor clinical prognosis	Articles reporting efficacy of MELK-targeted RNAi in cancer cell lines and/or xenograft tumor models	Articles reporting efficacy with small-molecule inhibitors of MELK in cancer cell lines and/or xenograft tumor models	Articles that made use of the MELK inhibitor OTS167 (also known as OTSSP167)
Gray et al. (2005)	x		x		
Lin et al. (2007)	x		x		
Marie et al. (2008)	x	x	x		
Nakano et al. (2008)	x	x	x		
Nakano et al. (2009)	x		x		
Pickard et al. (2009)	x	x	x		
Hebbard et al. (2010)			x		
Choi and Ku (2011)			x		
Chung et al. (2012)				x	x
Gu et al. (2013)	x	x	x		
Kuner et al. (2013)	x	x			
Minata et al. (2014)			x	x	
Wang et al. (2014)	x		x	x	x
Du et al. (2014)	x	x	x		
Alachkar et al. (2014)	x	x	x	x	x
Beke et al. (2015)				x	
Li et al. (2016)	x	x	x	x	x
Inoue et al. (2016)	x		x	x	x
Chung et al. (2016)				x	x
Kato et al. (2016)				x	x
Touré et al., 2016			x	x	
Wang et al. (2016)			x	x	x
Stefka et al. (2016)				x	x
Xia et al. (2016)	x	x	x		
Speers et al., 2016	x	x	x	x	x
Hiwatashi et al., 2016	x	x			
Kohler et al. (2017)	x	x		x	x
Simon et al. (2017)			x	x	x
Edupuganti et al. (2017)				x	
Liu et al., 2017	x	x	x	x	x
Matsuda et al. (2017)			x		
Bolomsky et al., 2017	x	x		x	x
Janostiak et al., 2017	x	x	x	x	x

Of those 33 MELK-cancer publications, 20 articles describe studies indicating that MELK expression is up-regulated (mostly based on RNA levels) in various human cancers, including ovarian, breast, NSCLC, SCLC, AML, prostate, pancreas, gastric, renal, astrocytoma, glioma, medulloblastoma, colorectal, liver, and rectal cancers (see column 2 in Table 1). Fifteen of the published MELK expression studies also report an association between increased MELK expression and poor clinical prognosis (see column 3 in Table 1). Significantly, 23 of the published MELK studies report efficacy of MELK-targeted RNAi in cancer cell lines and/or xenograft tumor models, thereby directly implicating MELK function in cancer cell proliferation or tumorigenic potential (see column 4 in Table 1). Consistent with those findings, 19 published studies describe efficacy with small-molecule inhibitors of the MELK kinase in cancer cell lines and/or tumor xenografts (see column 5 in Table 1). Notably, 15 of those studies made use of the MELK inhibitor OTS167 (also known as OTSSP167) that is currently being evaluated in human clinical trials, presumably prompted by many of these published pre-clinical findings (see column 6 in Table 1). In aggregate, these published studies would seem to make a compelling case for a likely role for MELK in a variety of human cancers.

However, in March 2017, in an article published in eLife, researchers at the Cold Spring Harbor Laboratory (CSHL) and Stony Brook University reported that they had used CRISPR/Cas9 technology to delete MELK in 13 different human cancer cell lines of various tissue origins, and that they had observed in no significant effects on proliferation (Lin et al., 2017). Notably, this analysis included several triple-negative breast cancer cell lines that, according to the 2014 eLife article by Wang et al., were MELK-dependent (based on the observed consequences of RNAi-induced MELK depletion). Lin et al. also noted that in several published whole-genome, unbiased RNAi studies to identify genes required by cancer cells, MELK dependency was never observed. Furthermore, they observed that cancer cells depleted of MELK by CRISPR editing were found to retain sensitivity to the MELK kinase inhibitor OTS167. This led Lin et al. to conclude that the observed cytotoxicity following OTS167 treatment reflected off-target, MELK-independent mechanisms, thus implying that the ongoing clinical development of OTS167 as a MELK-targeted therapeutic may be misguided. Notably, three other relatively recent publications have similarly concluded that OTS167 is likely to exhibit MELK-independent cytotoxic activity (Huang et al., 2017; Simon et al., 2017; Ji et al., 2016).

A number of the authors on the first of these articles (Huang et al., 2017) were also authors on the 2014 eLife article that implicated MELK as a therapeutic target in human cancer (Wang et al., 2014). However, the title of Huang et al. ("MELK is not necessary for the proliferation of basal-like breast cancer cells") confirmed the doubts that Lin et al. had raised about MELK as a target. Like Lin et al., the analysis by Huang et al. included CRISPR-mediated deletion of MELK in cancer cells, as well as the discovery of a novel potent and more MELK-selective inhibitor that failed to significantly affect the growth of putatively MELK-dependent cells. Huang et al. also employed a targeted protein degradation strategy to further support the conclusion that MELK is not a dependency gene in basal breast cancer. In addition, they analyzed various MELK-targeted shRNAs, including several that had been used in previous reports. This led to the conclusion that the differences in the findings reported by Wang et al. in 2014 and those reported by Huang et al. in 2017 were probably due to off-target effects, further highlighting the challenges involved in interpreting the results of RNAi studies in cancer cells.

Then, in early 2018, the CSHL group published a second article that refuted some of the other findings reported by Wang et al. in 2014 eLife article, and seemingly providing the final 'nail in the coffin' for MELK as a target for cancer drugs (Giuliano et al., 2018). In particular, they reported that overexpression of MELK failed to transform immortalized cells, as had been previously claimed. They also generated several additional MELK-deficient cancer cells using CRISPR technology and failed to observe any effects on growth in culture or in xenograft tumor models. Furthermore, Giuliano et al. used a highly selective MELK inhibitor to show that acute MELK inhibition did not affect the proliferation or anchorage-independent growth of several tested cancer cell lines previously reported to be MELK-dependent.

The definitive validation of most of the well-established therapeutic targets resulted from the collective efforts of many independent groups over many years.

So, what does this all mean for MELK as a therapeutic target? The three recent eLife articles certainly call into question many of the previous reports regarding MELK dependency in cancer cells. The numerous published RNA expression studies, which are less likely to be affected by technical artifacts, are probably valid overall. However, those findings might simply reflect the fact that MELK is a cell cycle-regulated gene (Badouel et al., 2010) that may appear to be upregulated in relatively rapidly growing cell populations, but which is not necessarily a 'driver' of tumorigenesis. It also remains possible that a role for MELK in cancer is highly context-dependent or that cancer cells with some degree of MELK dependency are able to adaptively 'rewire' to accommodate MELK disruption. While these are formal possibilities, the findings reported in the three recent eLife articles are likely to substantially diminish the enthusiasm for MELK as a therapeutic target.

These observations should serve as a stark reminder to the research community of the issues that arise when RNAi or pharmacologic tool compounds (in particular, small-molecule inhibitors) are used in studies that seek to validate candidate targets – even when numerous independent studies all reach similar conclusions. Moreover, the fact that cancer patients are currently being treated with OTS167 based on the published findings certainly highlights the importance of robust pre-clinical validation.

## Addressing the problem of target validation

How should journals deal with such a scenario? In recent years, the research community has become increasingly aware of the challenge of reproducibility in biomedical research. Notably, eLife is beginning to report findings from the Reproducibility Project: Cancer Biology, which is an ambitious effort to evaluate the scope of this problem in preclinical cancer research and to identify the factors that influence reproducibility more generally (Errington et al., 2014). And early results have already highlighted the challenges in robustly reproducing published findings.

Within the cancer research community, most investigators readily acknowledge that the validation of candidate targets is very challenging and is subject to a large number of variables that are difficult to adequately account for, even within a well-controlled experimental design (as described earlier). Moreover, these same investigators recognize that the definitive validation of most of the well-established therapeutic targets resulted from the collective efforts of many independent groups over many years, typically involving hundreds if not thousands of reported findings that were ultimately required to solidify the case.

Indeed, scientific discovery, in most cases, has historically been a self-correcting quest. Along the way, peer reviewers and journal editors do their best to ensure that published conclusions are supported by well-executed, scientifically-sound experimentation. And yet, we have a big problem. It is worth noting that, in our view, the authors of the early MELK validation articles listed in Table 1 were generally well intentioned, even if it appears, with the benefit of hindsight, that a number of the conclusions in these papers were mistaken. Whereas some might call for the retraction of all such articles – a logistically complicated undertaking to be sure – we favor approaches that promote the self-correction of target validation claims over time (possibly including the publication of formal corrections to some especially problematic articles) and reduce the frequency of flawed claims in new reports.

So how can we do better going forward? Considering that RNAi and pharmacologic tool compound reagents may be an especially significant source of erroneous findings, there could be substantial value in standardizing criteria for the acceptable use of these reagents prior to publication. For example, the development of publicly accessible databases that track the reliability and specificity of such reagents could be very useful. Along these lines, a consortium of investigators has recently described a community-driven wiki resource (http://www.chemicalprobes.org/) to help researches make the best use of available chemical probes (Arrowsmith et al., 2015). A similar resource for RNAi reagents is likely to have tremendous value for the community. Investigators would obviously benefit from such a resource, as would journal editors and reviewers because they could quickly check the validity of the critical reagents described in manuscripts as part of the peer review process.

As for previously published articles that report conclusions based on potentially problematic reagents – which would seem to include a significant number of the articles on MELK – it could be useful for journals to 'tag' those articles that have relied on reagents whose specificity has subsequently been called into question This would be useful information for any researchers considering the extension or replication of findings reported in these articles. It would undoubtedly require an organized effort from the research and publishing communities, and significant time, to widely implement such approaches – and hopefully, this is where we are heading. In the meantime, it remains incumbent on scientists, peer reviewers and journal editors to apply adequately rigorous standards to ensure that published claims have a very high likelihood of standing the test of time – especially those that relate to the validation of candidate targets for drugs that might be used to treat human disease.
